# *Helicobacter pylori* Counteracts the Apoptotic Action of Its VacA Toxin by Injecting the CagA Protein into Gastric Epithelial Cells

**DOI:** 10.1371/journal.ppat.1000603

**Published:** 2009-10-02

**Authors:** Amanda Oldani, Mireille Cormont, Veronique Hofman, Valentina Chiozzi, Olivier Oregioni, Alexandra Canonici, Anna Sciullo, Patrizia Sommi, Alessia Fabbri, Vittorio Ricci, Patrice Boquet

**Affiliations:** 1 Department of Physiology, Human Physiology Section, University of Pavia, Pavia, Italy; 2 INSERM Unit 895 (Group 7), Faculty of Medicine, Nice, France; 3 INSERM ERI-21, Faculty of Medicine, Nice, France; 4 Department of Clinical Bacteriology, Nice University Hospital, Nice, France; 5 Department of Drug Research and Evaluation, Istituto Superiore di Sanità, Roma, Italy; University of Illinois, United States of America

## Abstract

Infection with *Helicobacter pylori* is responsible for gastritis and gastroduodenal ulcers but is also a high risk factor for the development of gastric adenocarcinoma and lymphoma. The most pathogenic *H. pylori* strains (i.e., the so-called type I strains) associate the CagA virulence protein with an active VacA cytotoxin but the rationale for this association is unknown. CagA, directly injected by the bacterium into colonized epithelium via a type IV secretion system, leads to cellular morphological, anti-apoptotic and proinflammatory effects responsible in the long-term (years or decades) for ulcer and cancer. VacA, via pinocytosis and intracellular trafficking, induces epithelial cell apoptosis and vacuolation. Using human gastric epithelial cells in culture transfected with cDNA encoding for either the wild-type 38 kDa C-terminal signaling domain of CagA or its non-tyrosine-phosphorylatable mutant form, we found that, depending on tyrosine-phosphorylation by host kinases, CagA inhibited VacA-induced apoptosis by two complementary mechanisms. Tyrosine-phosphorylated CagA prevented pinocytosed VacA to reach its target intracellular compartments. Unphosphorylated CagA triggered an anti-apoptotic activity blocking VacA-induced apoptosis at the mitochondrial level without affecting the intracellular trafficking of the toxin. Assaying the level of apoptosis of gastric epithelial cells infected with wild-type CagA^+^/VacA^+^ *H. pylori* or isogenic mutants lacking of either CagA or VacA, we confirmed the results obtained in cells transfected with the CagA C-ter constructions showing that CagA antagonizes VacA-induced apoptosis. VacA toxin plays a role during *H. pylori* stomach colonization. However, once bacteria have colonized the gastric niche, the apoptotic action of VacA might be detrimental for the survival of *H. pylori* adherent to the mucosa. CagA association with VacA is thus a novel, highly ingenious microbial strategy to locally protect its ecological niche against a bacterial virulence factor, with however detrimental consequences for the human host.

## Author Summary

The gram-negative bacterium *Helicobacter pylori* is the main causative agent of peptic ulcer and gastric cancer in humans. Our work sheds light on a new molecular mechanism by which *H. pylori* would exert its highly efficient colonization strategy of the human host. In this paper, we show that the *H. pylori* CagA protein counteracts, by two distinct non-overlapping mechanisms, the apoptotic activity of the *H. pylori* VacA toxin on human gastric epithelial cells so as to allow a protection of the bacterium niche against VacA, giving a rationale for the association of these two virulence factors in the most pathogenic *H. pylori* strains. This is a new, highly ingenious mechanism by which a bacterium locally protects its ecological niche against the action of one of its own virulence factors. However, while exerting a beneficial role for survival and growth of the bacterium by counteracting VacA toxin, CagA injection in the gastric epithelial cells triggers proinflammatory and anti-apoptotic responses which are detrimental for the human host in the long-term and favor the development of ulcer and cancer.

## Introduction

Infection with *Helicobacter pylori* is responsible for gastritis and gastroduodenal ulcers but is also a high risk factor for the development of mucosa-associated lymphoid-like tissue (MALT) lymphoma as well as gastric adenocarcinoma [1]. *H. pylori* strains have been classified by the presence or the absence of two virulence factors, namely: an active vacuolating toxin VacA [2] and a 40-kbp pathogenicity island (*cag* PAI) encoding the 120–145 kDa immunodominant protein termed cytotoxin-associated gene A (CagA) as well as a type IV secretion system (TFSS) that injects CagA into the host cell [3]. Type I strains produce an active VacA and contain the *cag* PAI whereas type II synthesize an inactive VacA, due to mutations in the cytotoxin gene, and lack the *cag* PAI. *H. pylori* type I is responsible for the most severe pathogenic effects of the bacterium whereas type II induces mild gastritis only [4]. The CagA protein is probably the pivotal bacterial determinant for the development of a severe gastric inflammation that favors, in the long-term (years or decades), the occurrence of ulcer and cancer [5]. Albeit the VacA cytotoxin was first described as provoking cell vacuolation [6], it also induces apoptosis of gastric epithelial cells via the mitochondrial pathway (i.e., the so-called intrinsic pathway) [7]–[10]. As suggested [11], VacA may play a role in the early steps of bacterial gastric colonization.

Different host cell signaling cascades are activated by CagA [3],[12]. Importantly, different effects have been observed depending on the occurrence of phosphorylation by the Src kinase family (SKF) of tyrosine residues contained within EPIYA motifs located in the C-terminal 35–45 kDa part of CagA [3],[12]. A well-established response elicited by the phosphorylated form of CagA is a morphological process consisting in the formation of an elongated cell phenotype termed “hummingbird” [13]. This effect is mainly due to deactivation of the SKF by the tyrosine-phosphorylated CagA acting *in fine* on actin-binding proteins and producing a rearrangement of the actin cytoskeleton [14],[15]. In the unphosphorylated form, CagA stimulates the Erk1/2 pathway via its association with Grb2 [16] leading to a proinflammatory IL-8 response by activating the NF-kB pathway [17] and, as recently reported, antagonizing apoptosis of gastric cells at the level of the intrinsic pathway [18]. Quite recently, it has been reported that the phosphorylation-independent proinflammatory and anti-apoptotic activities of CagA could be accounted for by a C-terminal domain of CagA distinct from the EPIYA motif and named CRPIA (conserved repeat responsible for phosphorylation-independent activity) [19].

In the present study, we therefore examined whether CagA might be able to counteract the apoptotic effect of VacA. Here we demonstrate that, depending on tyrosine-phosphorylation by host kinases, CagA inhibited VacA-induced apoptosis by two complementary mechanisms. Tyrosine-phosphorylated CagA prevented pinocytosed VacA to reach intracellular compartments, a prerequisite for VacA-induced apoptosis. Unphosphorylated CagA triggered an anti-apoptotic activity blocking VacA-induced apoptosis at the mitochondrial level without affecting the intracellular trafficking of the toxin.

## Results

### The C-terminal signaling domain of CagA can mimick the phosphorylation-dependent and -independent activities of the full-length protein

**Figure 1:**
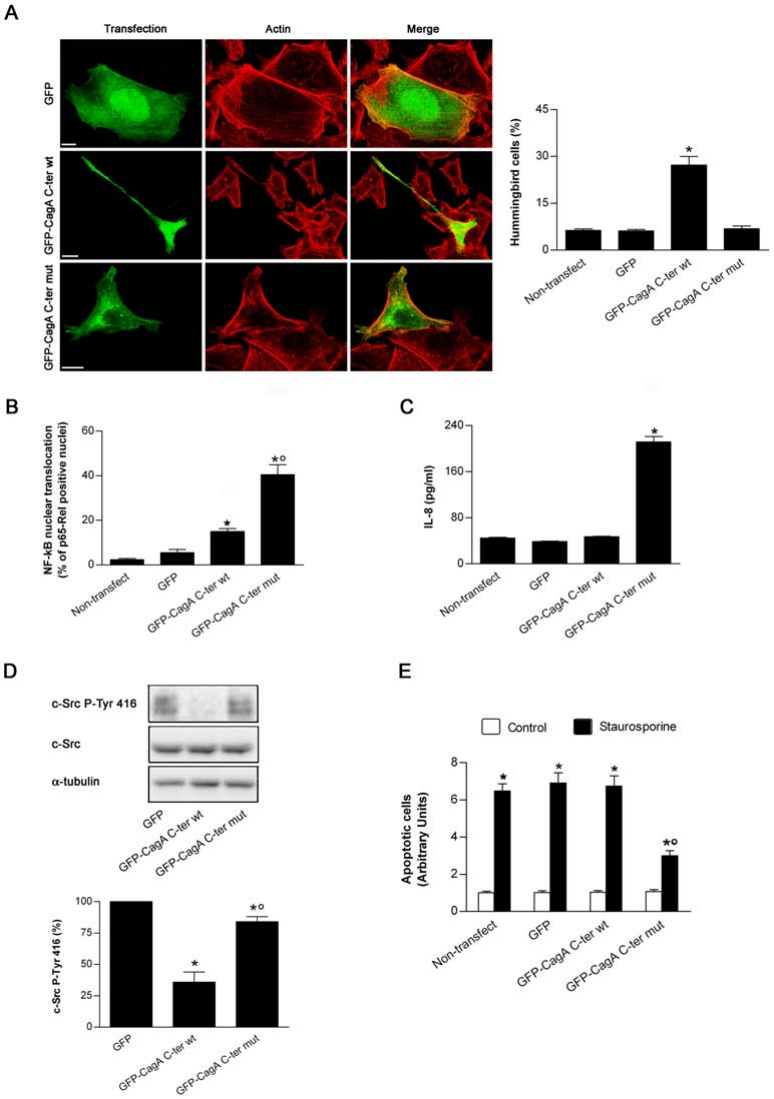
Effects of the GFP-CagA C-ter fusion proteins expressed into gastric epithelial cells. (A) Fluorescence of AGS cells transfected with GFP or GFP-CagA C-ter, either wt or mut. Transfected cells (green), actin staining (red). All the pictures shown represent single confocal sections. Scale bar: 10 µm. The graph: percentage of cells exhibiting the so-called “hummingbird phenotype” for each transfection condition. Mean±SEM by extensive confocal microscopy evaluation of slides from 3 independent experiments. *: *P*<0.05 versus non-transfected cells. (B) AGS cells transfected with GFP or GFP-CagAC-ter, either wt or mut, and then quantitatively analyzed for NF-kB p65-Rel nuclear translocation. Mean±SEM of 3 independent experiments. *: *P*<0.05 versus non-transfected cells. °: *P*<0.05 versus GFP-CagA C-ter wt. (C) AGS cells transfected with GFP or GFP-CagA C-ter, either wt or mut. After 24 h the cell culture supernatants were tested for IL-8. Mean±SEM of 3 independent experiments. *: *P*<0.05 versus non-transfected cells. (D) Representative blot (top) showing expression of c-Src, either total or activated form (c-Src P-Tyr 416; i.e., exhibiting tyrosine 416 phosphorylation) in AGS cells transfected with GFP or GFP-CagA C-ter, either wt or mut. Histograms (bottom) represent the quantitation of c-Src P-Tyr 416 (normalized for protein loading (α-tubulin) and shown as percentage of GFP-transfected control cells) for each transfection condition. Mean±SEM of 3 independent experiments. *: *P*<0.05 versus GFP. °: *P*<0.05 versus GFP-CagA C-ter wt. (E) Apoptosis (shown as fold increase over the non-transfected control cells) induced by 3 h treatment with 0.5 µM staurosporine in AGS cells transfected with GFP or GFP-CagA C-ter, either wt or mut. Mean±SEM of 3 independent experiments. *: *P*<0.05 versus paired control. °: *P*<0.05 versus all other staurosporine-treated conditions.

To dissect the possible molecular mechanisms by which CagA might interfere with VacA-induced apoptosis, we constructed fusion molecules with the GFP protein encompassing or not the 38 kDa C-terminal signaling domain of CagA at the C-terminus of GFP (GFP-CagA C-ter wt) (Figure S1A). The 38 kDa C-terminal part of the CagA from *H. pylori* strain 26695 [20] contains 2 consensus EPIYA motifs (named A and C, respectively) that may be tyrosine-phosphorylated by SKF [21]. In the present study we used only the C-terminal signaling part of CagA since, while the 100 kDa N-terminal domain of the protein is important for determining its intracellular localization, the CagA C-terminal domain is normally phosphorylated by SKF and fully able to reproduce the effects of phosphorylated full-length CagA independently of the presence of the N-terminal domain [22]. We reasoned that by preventing EPIYA phosphorylation through site-directed mutagenesis of its tyrosine residues we would preserve only the phosphorylation-independent effects of CagA. We thus mutated the tyrosine residues contained within the two EPIYA motifs into glycine (GFP-CagA C-ter mut) (Figure S1A). Then, we tested the effects of the wt and mut constructions in human gastric epithelial AGS cells. Upon transfection of these DNA constructs into cultured AGS cells, the resulting proteins were expressed at their expected molecular sizes. They reacted with both the anti-GFP and anti-CagA antibodies and exhibited a roughly similar repartition between membrane and cytosolic fractions (Figure S1B). The GFP-CagA C-ter was associated almost equally between the membrane and cytosolic fractions (differently from the GFP molecule alone that was mainly cytosolic) (Figure S1B). This is in agreement with the observation that a single EPIYA motif favors the recruitment of CagA to the plasma membrane, independently of its tyrosine phosphorylation [23]. As expected, the GFP-CagA C-ter wt was tyrosine-phosphorylated whereas the mutated form was not (Figure S1C). As previously shown [13],[22], the CagA C-ter wt greatly increased the number of cells showing the characteristic elongated hummingbird phenotype (Figure 1A), due to deactivation of SKF by the tyrosine-phosphorylated CagA [14],[15]. This effect was not observed in cells transfected with the GFP-CagA C-ter mut, albeit the fusion protein was expressed at a same level than the wt form in the cytosol (Figure 1A). In AGS cells transfected with the GFP-CagA C-ter wt, only a slight increase in the basal level of NF-kB p65-Rel nuclear translocation (Figure 1B) and no IL-8 chemokine response were observed (Figure 1C). This is in agreement with the previous finding that the CagA C-ter domain does not fully reproduce the phosphorylation-independent effects of the full-length CagA [22]. Conversely, when the GFP-CagA C-ter mut was transfected into cells, a massive translocation of NF-kB p65-Rel into the nucleus (Figure 1B) and a robust IL-8 response were observed (Figure 1C), as reported previously for CagA injected into gastric cells by the *H. pylori* TFSS [15]. This result validated the use of the GFP-CagA-C-ter mut construct to obtain the cell effects of CagA independent of its phosphorylation. Finally, we tested whether our CagA C-ter constructs were able to decrease the activation of the c-Src kinase as previously reported for CagA [14]. In AGS cells transfected with either the wt or the mut form of CagA C-ter, we measured the phosphorylation level of the tyrosine 416 of c-Src. Indeed, tyrosine 416 phosphorylation is required for the activation of the Src kinase [24],[25]. As shown in Figure 1D, transfection with GFP-CagA C-ter wt caused a profound decrease in c-Src tyrosine 416 phosphorylation, while the mut form was only slightly effective. This confirms that inhibition of the SKF by CagA is specifically induced by the phosphorylated form of CagA [14].

Altogether, these results show that in a tyrosine-phosphorylated form, the signaling domain of CagA produces the characteristic morphological cell effects and inhibition of SKF activity whereas in an unphosphorylated form it activates proinflammatory responses. This demonstrated that, upon transfection into gastric epithelial cells, the GFP-CagA C-ter constructions can separately mimick the reported effects of the full-length CagA molecule injected into cells by *H. pylori* TFSS [3],[12],[26]. Interestingly, CagA has been shown to induce an anti-apoptotic activity [18],[27] protecting against etoposide (an inducer of apoptosis acting *in fine* on the mitochondrial pathway) recently shown to be antagonized by CagA in a phosphorylation-independent fashion [18]. This fact raised the possibility that our CagA C-ter constructs might differently affect cell apoptosis. We thus next tested whether the GFP-CagA C-ter constructs might affect the mitochondrial apoptotic pathway antagonizing, for instance, the cell death induced by staurosporine, a drug specifically triggering the intrinsic cell death pathway [28]. As shown in Figure 1E, only the mut form of the GFP-CagA C-ter construct was able to down-modulate staurosporine-induced apoptosis.

### CagA, either tyrosine-phosphorylated or not, counteracts the apoptotic activity of VacA

**Figure 2:**
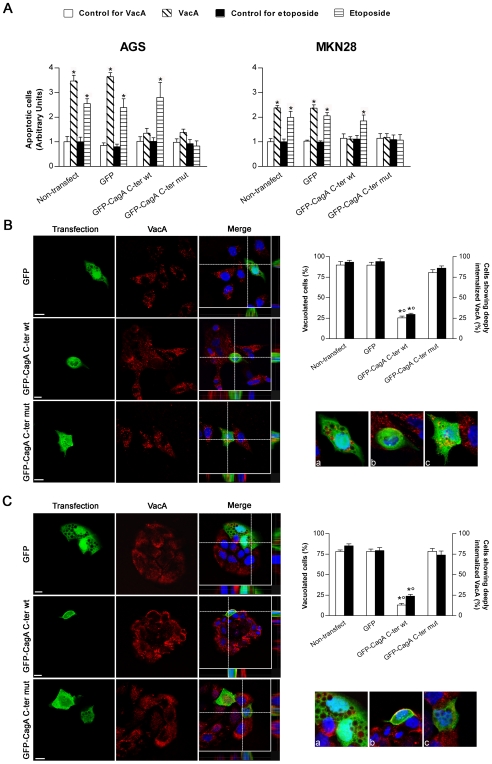
Transfection of gastric epithelial cells with GFP-CagA C-ter, either wt or mut, antagonizes VacA-induced apoptosis. (A) Apoptosis (shown as fold increase over the respective non-transfected control cells) induced by either VacA or etoposide in gastric AGS or MKN 28 cells. VacA was used at 5 µg/ml for 18 h, while etoposide at 300 µM for 6 h (AGS cells) or 24 h (MKN 28 cells). Mean±SEM of 3 independent experiments. *: *P*<0.05 versus paired control. (B and C) VacA internalization and its vacuolating action in transfected AGS (B) or MKN 28 (C) cells. VacA (red) and nuclei (blue). Transfected cells (green). All the pictures shown represent single confocal sections. Scale bar: 10 µm. For a more careful evaluation of VacA internalization, for each merged picture the overall z-stack profile of the cells along the depicted x-y axes were highlighted. (a), (b), and (c) are respectively zooms of the merged pictures at each x-y intersection. The graph (top right): percentage of either vacuolated cells (white columns) or cells deeply internalizing VacA (black columns) for each transfection condition. *: *P*<0.05 versus non-transfected cells. °: *P*<0.05 versus GFP-CagA C-ter mut.

We then investigated whether transfection of human gastric epithelial cells with either the wt or the mut forms of the GFP-CagA C-ter was able to antagonize the apoptotic activity induced by either the VacA cytotoxin or the drug etoposide. When non-transfected AGS or MKN 28 cells were treated with purified VacA, a statistically significant cell apoptosis was observed (Figure 2A). As expected, etoposide was also inducing a statistically significant apoptotic effect in both gastric cell lines (Figure 2A). In cells transfected with the GFP-CagA C-ter wt, the VacA-induced apoptotic effect was inhibited (Figure 2A). However, this CagA construction did not impair the apoptotic effect induced by etoposide (Figure 2A). In cells transfected with the GFP-CagA C-ter mut, the apoptotic effects induced by either VacA or etoposide were both inhibited (Figure 2A). Our results thus suggest that CagA may counteract VacA apoptotic activity by acting at two different molecular levels, one of which was clearly ineffective for etoposide. A possibility was that, in one hand, CagA might alter the effect of VacA by impairing the specific pinocytosis and/or intracellular trafficking of the cytotoxin [29],[30]. This mechanism would be clearly ineffective for etoposide that penetrates into cells by simple diffusion. On the other hand, the apoptotic activity of both VacA and etoposide might be antagonized by upregulation of anti-apoptotic factors since the two molecules act finally on the intrinsic cell death pathway.

### Tyrosine-phosphorylated CagA blocks the trafficking of VacA toward its target intracellular compartments

**Figure 3:**
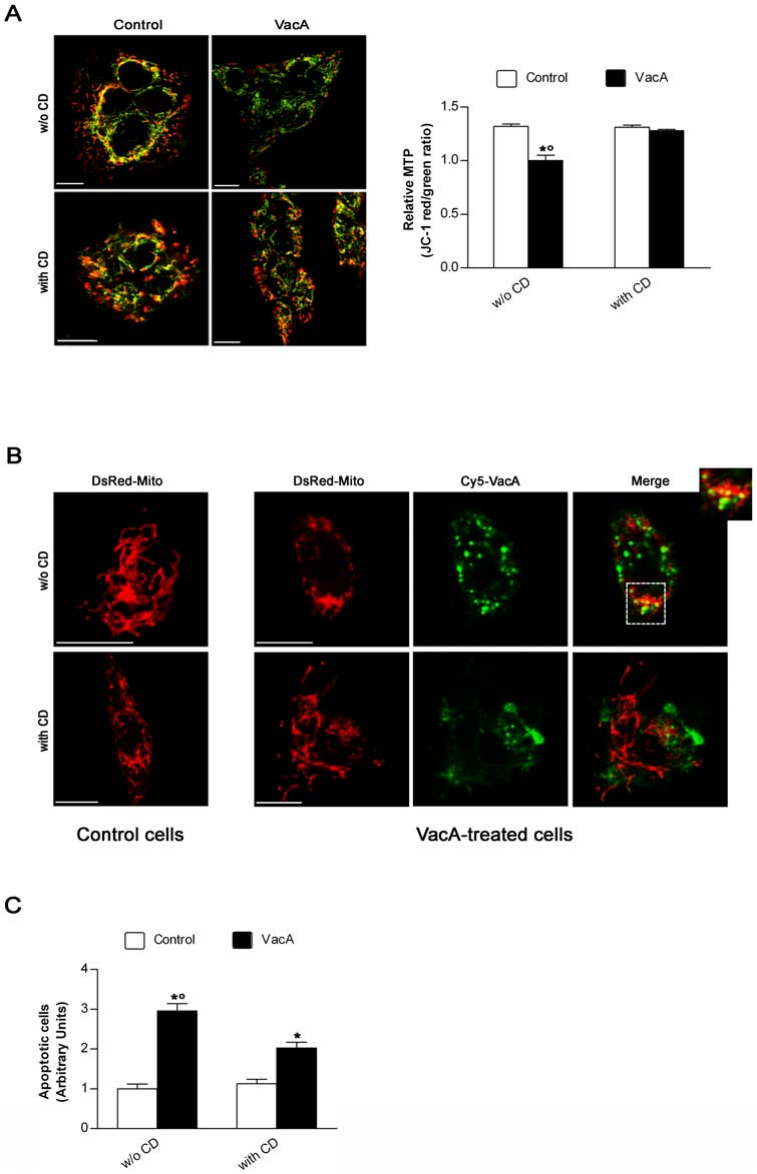
Cytochalasin D treatment protects gastric epithelial cells against both drop in mitochondrial transmembrane potential and apoptosis induced by VacA by preventing its mitochondrial localization. (A) Effect of cytochalasin D (CD) on mitochondrial transmembrane potential (MTP) of MKN 28 cells, treated or not with VacA, evaluated by using the ratiometric potential-sensitive dye JC-1 and confocal microscopy. All the pictures shown represent single confocal sections. Scale bar: 10 µm. The graph (right) shows the quantitation (mean±SEM) of relative MTP in the different experimental conditions by extensive confocal microscopy evaluation of slides from 3 independent experiments. *: *P*<0.05 versus paired control. °: *P*<0.05 versus the same condition in the presence of CD. (B) Morphology of mitochondria and their colocalization with VacA in AGS cells transfected with DsRed-Mito and treated or not with Cy5-labelled VacA in the absence or presence of CD. DsRed-Mito (red) and Cy5-VacA (green). All the pictures shown represent single confocal sections. Scale bar: 10 µm. (C) Apoptosis (shown as fold increase over the CD-untreated control cells) induced by VacA in MKN 28 cells treated or not with CD. Cells treated or not with CD but not exposed to VacA served as controls. Mean±SEM of three different experiments. *: *P*<0.05 versus paired control. °: *P*<0.05 versus the same condition in the presence of CD.

**Figure 4:**
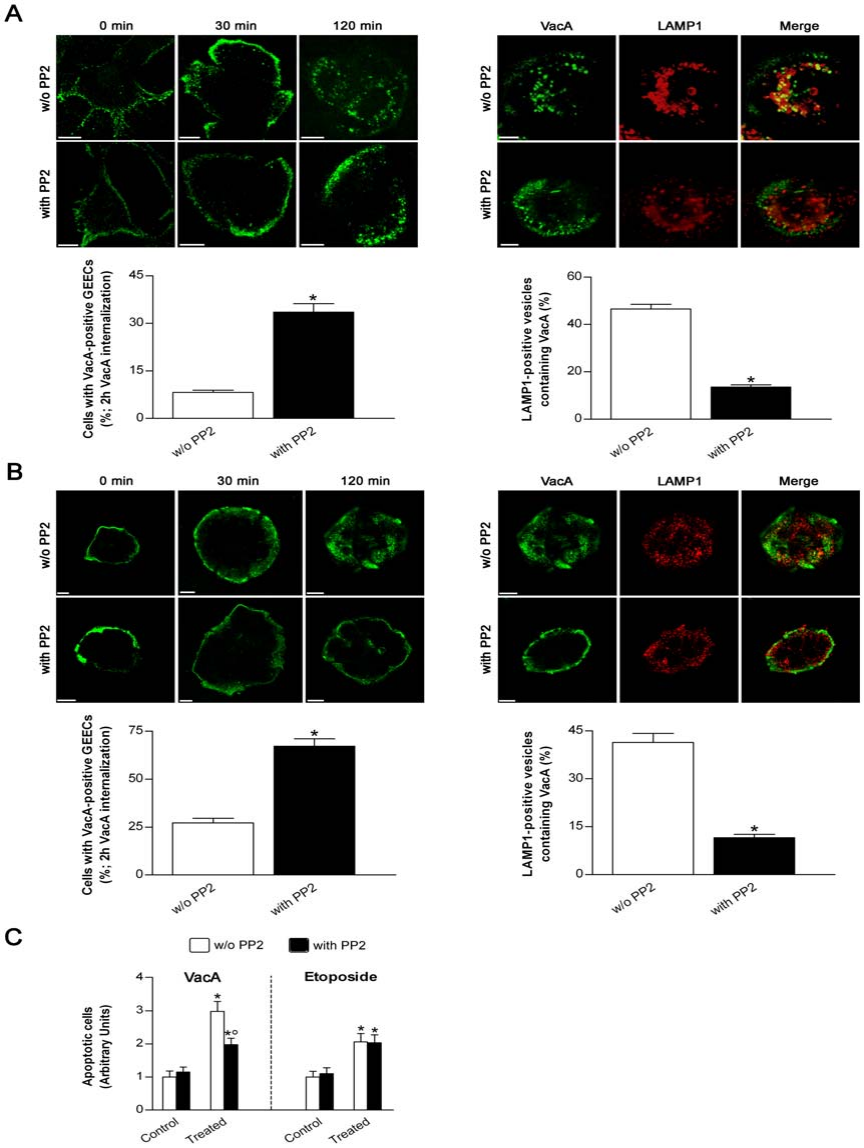
Treatment of gastric epithelial cells with the specific SKF inhibitor PP2 impairs the arrival of VacA into endosomes. (A and B) VacA intracellular trafficking in AGS (A) or MKN 28 (B) cells treated or not with PP2. Cells were preincubated for 30 min at 37°C in the presence or absence of 10 µM PP2 and, after a VacA binding step of 1 h at 4°C, immediately fixed (0 min) or allowed to internalize the toxin into early (30 min) or late (120 min) endocytic compartments (top left panels). VacA in green. For the 120 min time point, also LAMP1 (red; a marker of late endosomes) and its colocalization with VacA are shown (top right panels). All the pictures represent single confocal sections. Scale bar: 10 µm. The graphs (bottom panels): effect of PP2 treatment on either the percentage of cells exhibiting VacA confined in the GEECs (left) or the percentage of late endosomes (i.e., LAMP1-positive vesicles) containing VacA (right) after 2 h internalization. Mean±SEM by extensive confocal microscopy evaluation of slides from 3 independent experiments. *: *P*<0.05 versus PP2-untreated cells. (C) Apoptosis (shown as fold increase over the respective PP2-untreated control cells) induced by either VacA or etoposide in MKN 28 cells treated or not with PP2. Cells treated or not with PP2 but not exposed to VacA or etoposide served as controls. Mean±SEM of three different experiments. *: *P*<0.05 versus paired control. °: *P*<0.05 versus the same condition in the absence of PP2. The high cytotoxicity induced by PP2 on AGS cells after the incubation time used to measure either VacA- or etoposide-induced apoptosis did not enable us to repeat the same set of experiments in this latter cell line.

When the GFP-CagA C-ter wt was transfected into human gastric epithelial cells and then challenged with purified VacA, virtually no VacA was able to deeply penetrate inside the cells reaching intracellular compartments such as late endosomes (LEs; LAMP1-positive compartment) (Figure S2) and no vacuolation was observed in transfected cells compared to GFP-transfected or non-transfected control cells (Figure 2B and C). Clearly most of the toxin remained associated in the inner cell periphery, likely in the GPI-anchored-protein-enriched-early-endosomal-compartments (GEECs) (Figure S2 and zooms of Figure 2B and C), as we have previously shown [30]. This indicated that, after binding to its membrane receptor sphingomyelin [31], pinocytosis of VacA was not inhibited but the further intracellular trafficking of the cytotoxin was blocked. On the other hand, when cells were transfected with the GFP-CagA C-ter mut (whose cell expression pattern/localization was virtually identical to that of the wt form), both VacA in LEs and large vacuoles were easily observed, as in GFP-transfected or non-transfected cells (Figures S2 and 2B and C). Molecules pinocytosed into GEECs are transferred to early endosomes (EEs) [30]. We have previously shown that comet-like F-actin structures associated at the tip of EEs were required to sort out VacA from GEECs so as to transport the cytotoxin to LEs [32]. We made the hypothesis that also VacA-induced apoptosis could be provoked by the transport, mediated by F-actin motility of endosomes, of the toxin either directly to mitochondria [32] or to a specific intracellular compartment from which the toxin is then delivered into the cytosol to indirectly trigger the intrinsic cell death pathway as suggested [10]. We have shown that treatment of cells that have already pinocytosed VacA into GEECs (after 30 min of pinocytosis) with cytochalasin D (CD) prevented the cytotoxin to reach LEs, as shown by the inhibition of the vacuolation effect of VacA and the lack of colocalization of the cytotoxin with the late endosomal marker LAMP1 [32]. If VacA requires the same mechanism (i.e., actin-based motility of VacA-containing endosomes) to be transferred to mitochondria where it causes apoptosis, it should be possible to block the transfer of the cytotoxin to mitochondria and the following apoptosis by depolymerization of F-actin. Moreover, modification of the mitochondrial transmembrane potential (MTP) is an early event in VacA-induced apoptosis (contemporary to cell vacuolation) [33]. A mechanism likely provoked by the transfer of only very few toxin molecules into mitochondria [7],[34]. Using the same experimental approach previously described for CD-induced inhibition of VacA-induced vacuolation [32], we tested whether disruption of F-actin could affect the decrease in MTP and cell apoptosis induced by VacA as well as its trafficking to mitochondria. Using the ratiometric potential-sensitive dye JC-1 to evaluate the relative MTP, we found that CD treatment protected the gastric epithelial cells by VacA-induced decrease in MTP (Figure 3A). When DsRed-Mito-transfected cells were exposed to Cy5-VacA in the absence of CD treatment, we found that mitochondria lost their typical spaghetti-like morphology (which is becoming a *bona fide* marker for mitochodrial integrity [35],[36]) and showed some VacA molecules inside (Figure 3B). On the contrary, CD treatment was able to prevent both VacA-induced alteration in mitochondrial morphology and any colocalization of the toxin with mitochondria (Figure 3B). Furthermore, CD treatment caused a statistically significant decrease in VacA-induced apoptosis (Figure 3C). What might be the molecular mechanism by which CagA C-ter wt could block the trafficking of VacA-containing endosomes? It was recently shown that the Src-like kinase p61HcK was required to build comet-like F-actin structures at the tip of lysosomes [37] and tyrosine-phosphorylated CagA as been reported to inhibit the activation of the SKF [14],[15]. Accordingly, we found that cell transfection with the CagA C-ter wt construct caused a blockage of the phosphorylation level of the tyrosine 416 of c-Src (Figure 1D), which is essential for SKF kinase activity [25]. Using the specific SKF inhibitor PP2, we thus investigated whether deactivation of SKF could block the exit of the cytotoxin from GEECs by inhibiting the retrieval of VacA-loaded EEs from these compartments. Inhibition of the SKF in AGS or MKN 28 cells by PP2 considerably decreased the cytotoxin exit from GEECs to intracellular compartments such as LEs (LAMP1-positive compartment) (Figure 4A and B), as observed in GFP-CagA-C-ter wt transfected cells (zooms of Figure 3B and C). Furthermore, PP2 significantly decreased VacA-induced apoptosis (Figure 4C). On the contrary, PP2 was without any effect on etoposide-induced apoptosis (Figure 4C). These results highlighted the role of the SKF in the control of a specific step of VacA intracellular trafficking (i.e., its exit from GEECs) suggesting that one role of CagA (in its phosphorylated form) is to block, via SKF inhibition, the progression of pinocytosed VacA toward its target intracellular compartments (LEs and mitochondria).

### Unphosphorylated CagA does not increase the cell levels of Bcl2, Bcl-xL or Mcl1 although its anti-apoptotic action is completely mimicked by Bcl2 overexpression

**Figure 5:**
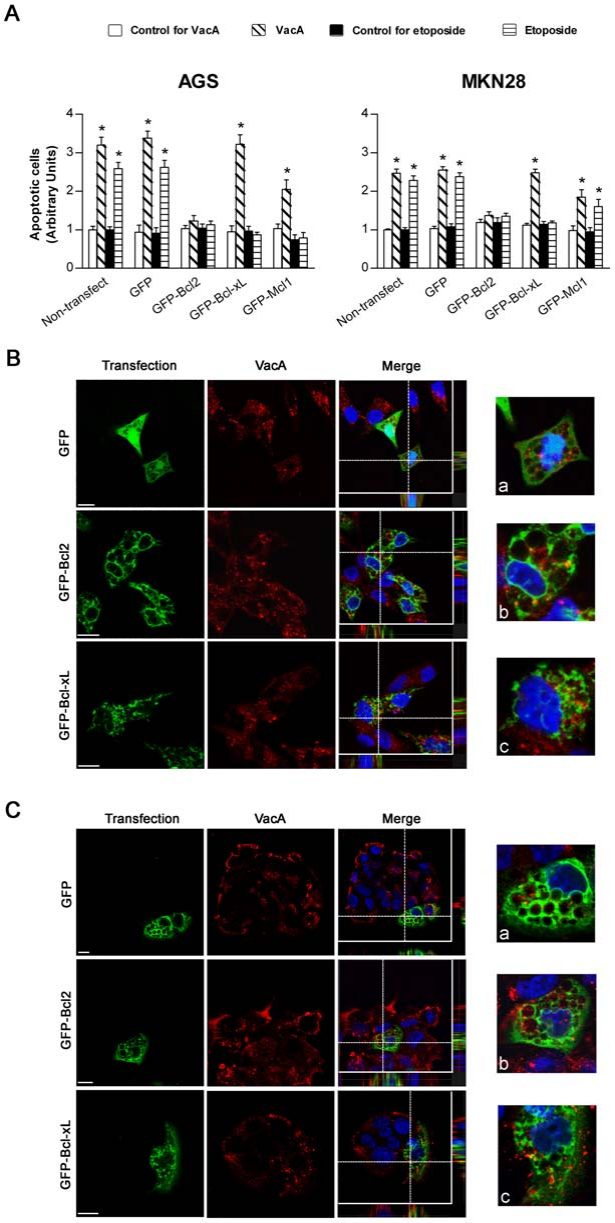
Transfection of gastric epithelial cells with GFP-Bcl2 inhibits VacA-induced apoptosis while not blocking pinocytosis or intracellular trafficking of the toxin. (A) Apoptosis (shown as fold increase over the respective non-transfected control cells) induced by either VacA or etoposide in AGS or MKN 28 cells transfected with GFP-Bcl2, GFP-Bcl-xL, GFP-Mcl1, or the GFP vector. Cells were treated with VacA or etoposide as in Figure 3A. Non-treated cells served as controls. Mean±SEM of 3 independent experiments. *: *P*<0.05 versus paired control. (B and C) VacA internalization in AGS (B) or MKN 28 (C) cells transfected with GFP-Bcl2, GFP-Bcl-xL, or the GFP vector. VacA (red) and nuclei (blue). Transfected cells (green). All the pictures shown represent single confocal sections. Scale bar: 10 µm. For a more careful evaluation of VacA internalization, in every merged picture the overall z-stack profile of the cells along the depicted x-y axes were highlighted. (a), (b), and (c) are respectively zooms of the merged pictures at each x-y intersection.

The second role of CagA (in its unphosphorylated form) consists in triggering an anti-apoptotic activity able to inhibit apoptosis induced not only by VacA but also by staurosporine (Figure 1E) and etoposide (Figure 2A). It has been shown that CagA may up-regulate the expression of anti-apoptotic factors of the Bcl2 family such as Bcl2 [27] or Mcl1 [18]. By transfecting gastric epithelial cells with plasmids encoding for GFP-Bcl2, GFP-Bcl-xL or GFP-Mcl1, we therefore tested the effects of these three different anti-apoptotic factors of Bcl2 family in the protection of VacA- or etoposide-induced apoptosis. We found that cells transfected with the GFP-Bcl2 construct were highly resistant to the apoptotic action of either the cytotoxin or etoposide (Figure 5A). This finding well fits with our previous demonstration that Bcl2 antagonized the apoptotic activity of VacA when the toxin was expressed into cells [7]. On the other hand, cells transfected with the GFP-Bcl-xL construct were not protected against the apoptotic effect of VacA whereas the cell death provoked by etoposide was blocked (Figure 5A). It is worth noting that either Bcl2 or Bcl-xL expression did not interfere with VacA endocytosis in AGS or MKN 28 cells as evaluated by confocal microscopy (Figure 5B and C). Finally, the GFP-Mcl1 construct induced a significant (even though partial) protection against VacA-induced apoptosis in AGS cells as well as in MKN 28 cells (Fig. 5A). In both cell lines tested GFP-Mcl1 overexpression afforded also a protective action (complete in AGS while only partial in MKN28 cells) against etoposide-induced apoptosis (Figure 5A). We thus tested whether the GFP-CagA C-ter wt or mut could induce an up- or down-regulation of these anti-apoptotic factors. However, we were unable to find a statistically significant increase or decrease in the expression of these factors by either the wt or mut form of the CagA C-ter (Figure S3).

### Infection of gastric epithelial cells with CagA-defective *H. pylori* confirms that CagA counteracts the apoptotic activity of VacA

**Figure 6:**
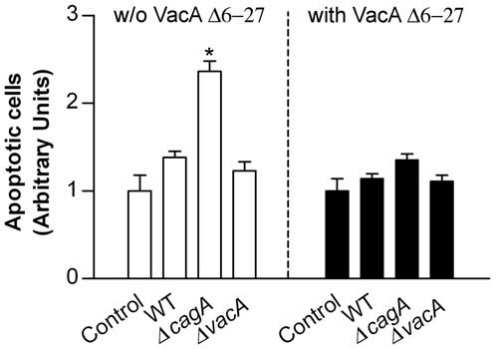
CagA antagonizes VacA-induced apoptosis in *H. pylori*-infected MKN 28 gastric epithelial cells. Apoptosis degree (shown as fold increase over the respective non-infected control cells) of MKN 28 cells infected with the wild-type CagA^+^/VacA^+^ *H. pylori* strain 60190 (WT) or its isogenic mutants lacking CagA (Δ*cagA*) or VacA (Δ*vacA*), respectively, in the absence or presence of 5 µg/ml dominant-negative purified VacA Δ6-27 cytotoxin. Mean±SEM of 3 independent experiments. *: *P*<0.05 versus paired control.

To confirm the results obtained in cells transfected with the CagA C-ter constructions showing that CagA antagonizes VacA-induced apoptosis, we finally measured the level of apoptosis of gastric epithelial cells infected with wild-type CagA^+^/VacA^+^ *H. pylori* or isogenic mutants lacking of either CagA or VacA. MKN 28 cells infected with the wild-type CagA^+^/VacA^+^ 60190 *H. pylori* strain showed an apoptosis degree slightly higher (even though not statistically significant) than that observed in non-infected control cells and virtually identical to that induced by its VacA-defective isogenic mutant (Figure 6). On the contrary, upon infection with the CagA-defective isogenic mutant, we observed an amount of cell apoptosis significantly higher than that caused by either the wild-type or the VacA-defective strain (Figure 6). The stronger apoptotic action exerted by the CagA-defective strain in comparison to its parental strain appeared completely due to its VacA production since, when infection of MKN 28 cells with *H. pylori* was carried out in the presence of an excess of the dominant-negative purified VacA Δ6-27 mutant cytotoxin [8],[38], we found an apoptosis level virtually identical for all the three strains tested (i.e., the wild-type and its isogenic mutants lacking CagA or VacA, respectively) (Figure 6). Indeed, VacA Δ6-27 is a well-known mutant toxin that not only lacks any vacuolating and apoptotic activity by itself but also, when mixed with wild-type VacA, specifically inhibits both cell vacuolation and apoptosis induced by the wild-type toxin [8],[38]. The anti-apoptotic activity of CagA in *H. pylori*-infected MKN 28 cells was further verified using another wild-type CagA^+^/VacA^+^ strain (i.e., G27) and its CagA-defective isogenic mutant (Figure S4). By using these latter *H. pylori* strains, the anti-apoptotic activity of CagA was also confirmed in *H. pylori*-infected AGS cells where, in agreement with previous findings [8], the wild-type CagA^+^/VacA^+^ strain was however causing a significantly higher degree of apoptosis in comparison to non-infected control cells (Figure S4).

## Discussion

In the present study we have shown that injection of the CagA protein by TFSS into gastric epithelial cells induced a protection against the apoptotic effects of the VacA cytotoxin. Importantly, depending on the tyrosine phosphorylation state of CagA, two separate mechanisms of protection were unraveled. However, because of the multifunctional activities of CagA [3],[12],[26], we cannot exclude that others CagA-triggered cell signaling pathways might be also involved in the protection against VacA-induced apoptosis. Indeed, mounting evidence suggests the existence of a substantial cross-talk between CagA and VacA in *H. pylori*-infected gastric epithelial cells. VacA might inhibit the activation of the transcription factor NFAT induced by CagA [39] or the cell scattering and elongation activities of CagA [40] which in turn might decrease the vacuolation induced by VacA [41]. Thus CagA and VacA appears to counteract their respective activities on host cells.

To study the possible activities of CagA on VacA-induced apoptosis, the experimental approach we used was based on i) cell transfection assays involving the expression of the C-terminal domain of the CagA protein that can be (GFP-CagA C-ter wt) or not (GFP-CagA C-ter mut) tyrosine phosphorylated by cellular SKF and ii) direct cell infection assays by wild-type *Helicobacter pylori* or its isogenic mutants defective in CagA or VacA. For transfection experiments, we used the C-terminal domain of CagA since it contains the essential signaling motifs that modulate the activities of the protein [19],[42],[43] and already shown to induce the effects of CagA [22]. Cell transfection experiments allowed us to dissect, at the molecular level, possible signaling pathways by which CagA affords protection against VacA-induced apoptosis.

In a first set of experiments, we validated our GFP-CagA C-ter cell transfection assays by showing that the signaling 38 kDa C-terminal domain of CagA containing the EPIYA motifs was indeed responsible for the important effects of CagA on host cells as previously reported by several groups [19],[22],[42],[43]. For instance, cell elongation (i.e., the hummingbird phenotype) [13],[42],[44],[45] is strictly phosphorylation-dependent whereas activation of NF-kB, production of IL-8 and protection against apoptosis are CagA activities independent of its tyrosine phosphorylation [16]–[19].

In cells transfected with the GFP-CagA C-ter wt, most of the VacA molecules were unable to deeply penetrate inside the cells but remained confined immediately under the plasma membrane, likely in GEEC compartments we have described previously [30]. This indicated that the first step of VacA endocytosis (namely pinocytosis) [30] was not affected by CagA. To leave GEECs and be able to traffick to internal cell compartments (i.e., LEs and mitochondria), vesicles containing VacA must be associated with dynamic actin structures [32]. We have previously shown that dynamic F-actin structures associated at the tips of EEs were required to sort VacA from GEECs to intracellular compartments [32], probably to retrieve VacA-loaded EEs out of GEECs and to allow trafficking to LEs [32]. Association of dynamic actin structures with EEs, required for the trafficking of ligands from EEs to LEs, has been recently confirmed [46]. Interestingly, formation of these dynamic actin structures are dependent upon the tyrosine phosphorylation of annexin 2 [47]. CagA wt decreases the tyrosine kinase activity of SKF ([14],[15], this study). Likely, the drop of SKF activity by the phosphorylated form of CagA blocked a critical step necessary for the motility of VacA-containing compartments to reach its intracellular targets. Using the pan SKF inhibitor PP2 we have shown that, as for the transfection of cells with the GFP-CagA C-ter wt, PP2 blocked the exit of VacA-containing vesicles from GEECs resulting in protection against apoptosis induced by the toxin. These findings indicated that VacA trafficking from GEECs to intracellular targets is controlled by SKF. Restriction of VacA to GEECs, by treatment with CD after the first step of cytotoxin endocytosis, not only decreased the cell vacuolation [32] but also both MTP drop and apoptosis induced by VacA (this study).

One study hypothesized that VacA may act only indirectly on mitochondria to release cytochrome *c* taking that i) isolated mitochondria incubated with VacA had a drop in the MTP but apparently no release of cytochrome *c*, and ii) in cells incubated with VacA the cytosolic proapoptotic factor Bax was activated while no colocalization between mitochondria and the toxin was found [10]. This result was challenged by the demonstration that isolated mitochondria incubated with VacA release a small but statistically significant amount of cytochrome *c* [48]. VacA or its p37 N-terminal domain are able to directly enter isolated mitochondria and to localize into the mitochondrial matrix/inner membrane and to induce apoptosis [7],[9],[34],[49]. Therefore, our findings suggest that, as in the case of other potent bacterial toxins acting intracellularly [50], only a small proportion of pinocytosed VacA reach its final target (i.e., mitochondria) albeit a large amount of the cytotoxin is routed to LEs.

For either the direct or indirect activation of the cell death intrinsic pathway, cytochrome *c* release by mitochondria requires the permeabilization of the mitochondrial outer membrane (MOM) by oligomerization of proapoptotic proteins of the Bcl2 family (Bax and Bak) [51],[52]. Thus it is not surprising to observe activation of Bax (or Bak) during that process [10]. Importantly, a direct activity of VacA on mitochondria for toxin-induced cell death is supported by the observation that mutations of the N-terminal p37 domain of VacA which blocks the transfer of the toxin into mitochondria also impairs VacA-induced apoptosis [7]. Therefore, a possible mechanism for VacA-induced mitochondrial damage could be the following: via endosomes propelled by actin polymerization, VacA is transferred to mitochondria (either directly by membrane-to-membrane contacts or after release into the cytosol followed by importation into mitochondria) where full-length VacA or its p37 N-terminal domain translocates to the mitochondrial inner membrane (MIM)/matrix of the organelle [7],[9]. There, VacA (or its N-terminal p37 domain) induces MIM depolarization, for instance by making a channel in the MIM as proposed recently [9],[34]. This activity might disrupt mitochondrial cristae junctions by interfering with the MIM OPA1 protein or molecules interfering with the control of OPA1, a critical step of cytochrome *c* release [53]. This may allow the release of large quantities of cytochrome *c* in the intermembrane mitochondrial space and, due to a small leakage of the MOM (which is not a fully impermeable membrane), a small amount of cytochrome *c* could be released out of mitochondria as observed in vitro [48]. This small release of cytochrome *c* might be able by itself to trigger the activation of a minute amount of caspase 3. Since caspase 3 has been reported to induce Bax translocation to MOM amplifying the release of cytochrome *c* [54], the initial direct effect of VacA on MIM would be followed by a massive, Bax-dependent, release of cytochrome *c*.

We have shown that in its unphosphorylated form CagA was able to induce an anti-apoptotic response not only toward staurosporine or etoposide as already described [18],[19], but also against VacA. The Bcl2 anti-apoptotic factor, but not the Bcl-xL or Mcl1 factors, has been reported to be upregulated by CagA [27]. More recently, the anti-apoptotic factor Mcl1 has been shown to be upregulated, through activation of the NF-kB pathway, specifically by the unphosphorylated form of CagA [18],[19]. In the present study we have been unable to demonstrate that the C-ter domain of CagA might significantly up- or down-regulate Bcl2, Bcl-xL or Mcl1. Nevertheless, overexpression of Bcl2 and, although to a lesser extent, Mcl1 clearly afforded a protection against VacA-induced apoptosis in both AGS and MKN28 cells mimicking the action of unphosphorylated CagA. Interestingly, it has been quite recently reported that, in nonmyeloid cells, *Shigella flexneri* was able to induce mitochondrial-dependent cell death but, at the same time, the bacterium was also triggering a prosurvival Nod1-dependent Rip2/IKKβ/NF-kB signaling pathway [55]. However, activation of the NF-kB pathway was not acting by increasing the expression level of Bcl2 family members, but rather preventing Bcl2 expression drop during bacterial infection [55]. A similar effect might take place during *H. pylori* infection and Bcl2 and Mcl1 might be the anti-apoptotic factors involved in CagA action against VacA-induced apoptosis.

It is well known that CagA is rapidly tyrosine phosphorylated after its injection into host cells [3],[12],[26]. At difference, production of IL-8 by the unphosphorylated form of CagA appears to be a very late event (starting after 24 h of infection) upon cell infection by *H. pylori* [17]. Therefore, the earliest mechanism for CagA-dependent inhibition of VacA-induced apoptosis would likely rely on the activity of the phosphorylated form of CagA. Indeed, blocking the trafficking of VacA would be an efficient mechanism for its neutralization. In agreement with that possibility, it has been demonstrated that injection of CagA into cells was strongly decreasing the vacuolating activity of the toxin [41]. Vacuolation induced by VacA is an early event and depends on the transfer of the cytotoxin to LEs. In the present study, we have shown that the phosphorylated form of CagA was indeed blocking the trafficking of the pinocytosed VacA and reduced the cell vacuolation induced by the cytotoxin. We have here also shown that blocking the motility of VacA-containing endosomes also decreased both MTP drop and apoptosis provoked by VacA and, interestingly, abolished the colocalization of the cytotoxin with mitochondria. These observations indicate that both the trafficking to LEs and to mitochondria necessitates an actin-driven vesicle activity [32]. However, even if the large majority of pinocytosed VacA is prevented to reach its intracellular targets by the protection pathway dependent on tyrosine-phophorylated CagA, also a very small amount of cytotoxin molecules reaching mitochondria could be enough to significantly activate the intrinsic apoptotic pathway during the course of infection. Apoptosis could be efficiently counteracted by activation of the NF-kB pathway allowing the upregulation of anti-apoptotic factor(s) (together with the production of IL-8). IL-8 production, a bystander of the NF-kB activation triggered by unphosphorylated CagA, is detectabled only after 24 h of infection [17]. This suggests that this kind of CagA-dependent protection against VacA-induced apoptosis is probably a later event during cell infection by *H. pylori*. Thus the combination of an earlier event (due to phosphorylated CagA), preventing most VacA molecules to reach mitochondria (and LEs), with a later event (due to unphosphorylated CagA), directly blocking the intrinsic apoptotic pathway, would constitute a clever and highly efficient system for protecting the cells against VacA-induced apoptosis.

Apparently, the VacA toxin plays a role during *H. pylori* stomach colonization [11]. However, once bacteria have colonized the gastric niche, the apoptotic action of VacA might be detrimental for the survival of *H. pylori* adherent to the mucosa. By injecting CagA into the colonized host cells, protection against VacA apoptosis would be afforded with great efficiency. In addition, as recently proposed, the anti-apoptotic activity of CagA might also dampen epithelial gastric cell renewal [18] whereas VacA would decrease the CagA-induced cell scattering and motility [40]. Interestingly, in this scenario the VacA toxin could still diffuse at distance from the bacteria colonizing the surface gastric epithelium to decrease, for instance, both the innate and adaptive immune response of the host against *H. pylori* [56]. This might finally explain the virtually constant association of an active VacA toxin with CagA in highly pathogenic *H. pylori* strains. CagA association with VacA is thus a novel, highly ingenious microbial strategy to locally protect its ecological niche against a bacterial virulence factor, with however detrimental consequences for the human host. Indeed, by exerting proinflammatory and anti-apoptotic activities, CagA favors, in the long-term, the occurrence of the most severe gastric diseases such as peptic ulcer and gastric cancer.

## Materials and Methods

### Bacteria, VacA purification, human gastric epithelial cells, antibodies, and reagents

The *H. pylori* strain 26695 [20] was a gift of R. Haas (München, Germany). The wild-type CagA^+^/VacA^+^ *H. pylori* strain 60190 (ATCC 49503) and its isogenic mutants in which *cagA* (60190:M22) or *vacA* (60190:v1) genes were disrupted by insertional mutagenesis [57],[58] were kindly provided by T.L. Cover (Nashville, TN). The wild-type CagA^+^/VacA^+^ *H. pylori* strain G27 and its isogenic mutant (G27Δ*cagA*) in which *cagA* gene was disrupted by insertional mutagenesis [59] were kindly provided by R. Zarrilli (Naples, Italy). Bacteria were grown in Brucella broth medium (Difco, Detroit, MI) supplemented with 1% Vitox (Oxoid, Basingstoke, UK) and 5% heat-inactivated fetal bovine serum (FBS, from Cambrex BioScience, Verviers, Belgium) at 37°C under microaerobic conditions and continuous shaking [29].

VacA was purified from broth culture supernatant of wild-type *H. pylori* 60190 strain by ammonium sulphate precipitation and gel filtration chromatography using a Superose 6 10/300 GL column (Amersham Biosciences, Umea, Sweden) in accordance with Cover *et al.* [60] and its concentration was determined using the DC protein assay kit (Bio-Rad, Hercules, CA). Purified VacA was stored in melting ice and, immediately before use, was activated by dropwise acidification to pH 3.0 with 0.2 N HCl [29],[60]. For some experiments, purified VacA labeled with Cy5 dye was prepared and used as previously described [32]. The mutant VacA toxin (VacA Δ6-27), lacking a critical hydrophobic region near the VacA N-terminus and exhibiting a dominant-negative effect on both cell vacuolation and apoptosis induced by wild-type VacA [8],[38], was a kind gift of T.L. Cover.

Human gastric epithelial AGS (ATCC CRL-1739) or MKN 28 cells [61],[62] were grown in DMEM supplemented with 10% FBS and 200 mM glutamine or in DMEM/Ham's nutrient mixture F-12 (1∶1) (Sigma-Aldrich, St. Louis, MO) supplemented with 10% FBS, respectively, at 37°C in a humidified atmosphere of 5% CO_2_ in air. Cell transfection with the plasmids used in this study was carried out according to the manufacturer's instructions using FuGene6 (Roche Diagnostics, Mannheim, Germany) or Lipofectamine (Invitrogen, Carlsbad, CA) for AGS cells and DOTAP (Roche Diagnostics) for MKN 28 cells. For the experiments assaying CagA-induced cell production of anti-apoptotic factors, AGS cells were transfected by using the Amaxa electroporation technology (Lonza, Walkersville, MD) in order to maximize transfection efficiency.

The following primary antibodies were used: a) mouse monoclonal anti-GFP (clone 7.1), from Roche; b) polyclonal rabbit anti-CagA, kindly provided by A. Covacci (Siena, Italy); c) mouse monoclonal anti-phosphotyrosine (clone 4G10), from Euromedex (Souffelweyersheim, France); d) rabbit polyclonal anti-VacA IgG 958, kindly given by T.L. Cover; e) mouse monoclonal anti-LAMP1, from BD Biosciences (San Jose, CA); f) rabbit polyclonal anti-p65-Rel, from Santa Cruz Biotechnology (Santa Cruz, CA); g) mouse monoclonal anti-Bcl2 and rabbit polyclonal anti-Bcl-xL and anti-Mcl1, all from Santa Cruz Biotechnology; h) rabbit polyclonal against c-Src (from Santa Cruz), and phosphospecific rabbit polyclonal against c-Src phosphorylated at Tyr416 (Cell Signaling Technology, Danvers, MA); i) mouse monoclonal anti-α-tubulin, from Sigma-Aldrich. As secondary antibodies, we used: a) anti-mouse and anti-rabbit conjugated to horseradish peroxidase (HRP), from Dako (Glostrup, Denmark); b) anti-mouse labeled with Alexa 488 (from Molecular Probes, Eugene, OR) or with aminomethylcoumarin acetate (AMCA; from Jackson Immunoresearch, West Grove, PA); c) anti-rabbit labeled with tetramethylrhodamine isothiocyanate (TRITC) or Cy5, from Jackson Immunoresearch. TRITC-labeled phalloidin and R-phycoerythrin-labeled (RPE) annexin V were purchased from Molecular Probes. Nuclear counterstaining was made with Hoechst 33258 (Sigma-Aldrich). The inhibitor of the Src family tyrosine kinases 4-amino-5-(4-chlorophenyl)-7-(*t*-butyl)pyrazolo[3,4-d]pyrimidine (PP2) and the apoptosis-inducer etoposide were purchased from Biosource (Camarillo, CA) and Sigma-Aldrich, respectively.

### Construction of CagA fusion proteins and of site-directed mutations

The *cagA* 3′ region (940 bp), corresponding to the CagA protein C-terminal domain of 38 kDa, containing the two tyrosine phosphorylation motifs EPIYA, was amplified by Polymerase Chain Reaction (PCR) from the DNA of *H. pylori* 26695 strain using the primers CagA C-ter wt 5′ and CagA C-ter wt 3′ (see Table S1). The PCR product was cloned into the PCR2.1 vector (TOPO Cloning Kit; from Invitrogen) according to the manufacturer's instructions. The PCR product was then subjected to BSRG1/Not1 cleavage and subcloned into a modified pEGFP-N1 vector (Clontech Laboratories, Mountain View, CA) to produce fusion of CagA C-ter wild-type at the C-terminus of GFP (GFP-CagA C-ter wt). Mutations of the tyrosine residues 899 and 972 into glycine to produce GFP-CagA C-ter mut were performed using the QuikChange site-directed mutagenesis kit (Stratagene, La Jolla, CA) according to the manufacturer's instructions with the oligonucleotides described in Table S1.

### Immunoprecipitation, immunoblotting, cell fractionation, actin staining, NF-kB nuclear translocation, IL-8 chemokine production, and expression of Bcl2, Bcl-xL, and Mcl1

For immunoprecipitation of tyrosine-phosphorylated CagA C-ter fusion proteins, transfected AGS cells were first washed twice with a cold (4°C) “stop buffer” (20 mM Tris-HCl, pH 7.5, containing 150 mM NaCl, 100 mM NaF, 2 mM Na_3_VO_4_, 10 mM Na_4_P_2_O_7_, 10 mM EDTA) and then scraped off from the culture dish in lysis buffer (LB) (20 mM Tris-HCl, pH 7.4, containing 150 mM NaCl, 100 mM NaF, 2 mM Na_3_VO_4_, 10 mM Na_4_P_2_O_7_, 10 mM EDTA, 1 µM okadaic acid, 1% Triton X-100, and a protease inhibitor cocktail (Complete EDTA-free, Roche)). Cells were then lysed in LB for 45 min at 4°C under constant agitation. Cell lysates were centrifuged at 10,000 *g* for 5 min and protein concentration in the supernatant was measured. Anti-phosphotyrosine antibodies were adsorbed on A/G sepharose beads (Tebu-Bio, Le Perray en Yvelines, France) under agitation and then added to 0.5 to 1 mg of cell supernatant proteins. Tyrosine-phosphorylated proteins were immunoprecipitated for 3 h at 4°C under constant agitation. Immunoprecipitated proteins were resuspended in a modified Laemmli buffer (100 mM Tris-HCl, pH 6.8, 0.2% bromophenol blue, 20% glycerol, 1.25% β-mercaptoethanol, and 4% SDS), resolved by SDS-PAGE, immunoblotted and detected by incubation of the membranes with anti-GFP or anti-CagA antibodies (dilution: 1∶500) followed by incubation with either anti-mouse or anti-rabbit HRP-conjugated antibodies. Immunoreactive bands were then revealed by enhanced chemiluminescence reaction (ECL, Amersham Biosciences). Cytosolic and membrane fractionation of transfected AGS cells was performed essentially as described by Stein *et al.* [63].

For actin staining and NF-kB nuclear translocation, variously transfected AGS cells grown on coverslip were incubated for 18 h, then fixed in 4% paraformaldehyde and permeabilized with 0.5% Triton X-100 in PBS. After washing, cells were incubated for 30 min at room temperature with TRITC-labelled phalloidin to stain F-actin or with the anti-p65-Rel antibody (dilution: 1∶50) followed by TRITC-labeled secondary antibody. After mounting in PBS/glycerol (1∶1, vol/vol), slides were analyzed with an Olympus BX51 fluorescence microscope.

For IL-8 chemokine production, incubation medium of variously transfected AGS cells was harvested 24 h after transfection as described by Kim *et al.* [64]. Samples were diluted 10 folds in culture medium and aliquots of 100 µl were assayed for IL-8 using the Flexia IL-8 ELISA kit (Biosource) as previously described [65]. In each experiment, transfections were made in triplicate and IL-8 assays in quadruplicate.

For Bcl2/Bcl-xL/Mcl1 expression analysis, after 24 h of incubation, variously transfected AGS cells were recovered in 20 mM Tris-HCl buffer (pH 7.4) containing 10 mM EDTA, 137 mM NaCl, 2 mM Na_3_VO_4_, 100 mM NaF and a protease inhibitor cocktail (Complete EDTA-free, Roche). Thirty µg of total proteins were separated by SDS-PAGE, transferred to a PVDF membrane and incubated with the indicated antibodies. Anti-mouse or anti-rabbit HRP-conjugated secondary antibodies were then added and chemiluminescence was detected by using the FUJIFILM Las-3000 apparatus. The same membrane was re-probed with anti-α-tubulin antibodies as a loading control. Signal quantification was performed using the MultiGauge software, and protein expression was normalized by that of α-tubulin.

### Confocal microscopy analysis

Immunofluorescence studies and analysis by confocal microscopy were performed as previously described [29],[30],[32] using a confocal laser scanning microscope (TCS SP2, from Leica, Heidelberg, Germany, or LSM510 Meta, from Carl Zeiss MicroImaging, Jena, Germany) equipped with 63× and 40× oil-immersion objectives. Briefly, variously transfected and treated cells grown on coverslip were fixed with 4% paraformaldehyde, permeabilized with 0.1% Saponin (Sigma-Aldrich) and processed for immunofluorescence. The images were combined and merged using Paint Shop Pro software (Corel, Ottawa, Canada). Quantitative evaluation (percentage) of cells showing the “hummingbird phenotype” under different transfection conditions was carried out by extensive evaluation of slides from 3 independent experiments. We defined a hummingbird cell that with an elongated phenotype characterized by thin needle-like protrusions longer than 10 µm [40],[44],[45]. For studying VacA endocytosis, cells were incubated with 2 µg/ml acid-activated purified VacA for 1 h at 4°C. After extensive washing, cells were incubated at 37°C in pre-warmed DMEM w/o FBS (for AGS cells) or Hanks' balanced salt solution (HBSS; for MKN 28 cells) for 30 or 120 min in the absence of ammonium chloride or, to allow vacuole development, for 4 h in the presence of 5 mM ammonium chloride. Cells were then processed for immunofluorescence analysis. All the pictures shown represent single confocal sections. When the effect of the SKF inhibitor PP2 on VacA internalization and intracellular trafficking was investigated, cells were preincubated with 10 µM PP2 at 37°C for 30 min before being exposed to VacA as described above, and the drug was maintained throughout the experiments. To investigate VacA internalization and intracellular trafficking in each transfection condition, by means of extensive confocal microscopy analysis of slides from at least 3 different experiments we calculated the percentage of either cells exhibiting cytoplasmic vacuoles (a widely accepted functional readout for the presence of VacA in the late endosomal compartment) or cells exhibiting the VacA-associated fluorescence strictly limited at the cell periphery (i.e., likely in the GEECs only) or, on the contrary, in the perinuclear area (i.e., cells deeply internalizing VacA). In addition, by means of colocalization and cell counter plugins of ImageJ software (National Institutes of Health, Bethesda, MD), in some experiments VacA intracellular trafficking to LEs was evaluated by calculating, in variously treated or control cells, the percentage of LAMP1-positive vesicles (i.e., LEs) containing VacA. When the effect of CD treatment on VacA trafficking to mitochondria and on toxin-induced alteration of mitochondrial morphology, DsRed-Mito-transfected AGS cells were incubated with 2 µg/ml activated Cy5-VacA for 1 h at 4°C. After washing, cells were allowed to internalize the toxin into GEECs by incubation for 15 min at 37°C in DMEM w/o FBS (and without ammonium chloride supplementation), then CD (0.5 µg/ml) was added or not and the cells were further incubated for 3 h. After fixation, cells were observed at the confocal microscope. To investigate the effect of CD treatment on VacA-induced MTP drop, MKN 28 cells grown on glass-bottomed dishes were incubated with VacA (2 µg/ml) for 15 min at 37°C to allow toxin entry into GEECs, washed and incubated for further 105 min with HBSS (without ammonium chloride supplementation) in the absence or presence of CD (0.5 µg/ml). Relative MTP was then measured by using the ratiometric potential-sensitive dye JC-1 (5,5′,6,6′-tetrachloro-1,1′,3,3′-tetraethylbenzimidazolylcarbocyanine iodide; from Molecular Probes). Indeed, this cationic lipid exhibits potential-dependent accumulation in mitochondria, indicated by a fluorescence emission shift from green to red [66]. Mitochondrial depolarization is indicated by a decrease in the red/green fluorescence intensity ratio which depends only on MTP and not on other factors such as mitochondrial size, shape and density. JC-1 (6 µM) was added to the cells during the last 15 min of incubation and, after washing, cells were immediately analyzed at the confocal microscope. In each experiment, the red/green fluorescence intensity ratio was calculated for at least 100 cells for each experimental condition by using the Leica software.

### Apoptosis assays

To study the apoptosis degree induced by either VacA or etoposide on variously transfected gastric epithelial cells, AGS cells grown in Petri dishes were treated with either 5 µg/ml acid-activated purified VacA for 18 h or 300 µM etoposide for 6 h in DMEM w/o FBS, while MKN 28 cells were treated with the aforementioned amounts of VacA or etoposide in HBSS for 18 h or 24 h, respectively. No supplementation of incubation medium with ammonium chloride was made. When the effect of CD treatment on VacA-induced apoptosis was investigated, MKN 28 cells were incubated with VacA (2 µg/ml) for 15 min at 37°C to allow toxin entry into GEECs, washed and incubated for further 18 h with HBSS in the absence or presence of CD (0.5 µg/ml). Then the incubation medium was collected and the adherent cells were splitted by means of TrypLE Express (Invitrogen) and added to the corresponding incubation medium. After centrifugation at 150 *g* for 5 min, the cell pellet was resuspended in 100 µl of binding buffer (10 mM Hepes, 140 mM NaCl, 2.5 mM CaCl_2_, pH 7.4) containing RPE-annexin V and incubated at room temperature for 15 min. Counting of the percentage of apoptotic (i.e., annexin V-positive) cells for each experimental condition was then carried out using a wide-field fluorescence microscope equipped with the proper filter sets. The percentages of apoptotic cells were shown as fold increase over the respective non-transfected control cells.

To study the apoptosis degree of gastric epithelial cells infected with wild-type CagA^+^/VacA^+^ *H. pylori* strains or their isogenic mutants lacking CagA or VacA, cells grown on Petri dishes were infected with the different *H. pylori* strains at a multiplicity of infection of 100 bacteria per cell and incubated in HBSS (for MKN 28 cells) or DMEM w/o FBS (for AGS cells) for 18 h at 37°C in the absence or presence of 5 µg/ml acid-activated purified dominant-negative VacA Δ6-27 cytotoxin added immediately before the bacteria. No supplementation of incubation medium with ammonium chloride was made. After the incubation period, cells were harvested and stained using the annexin V-FLUOS kit (Roche Diagnostics) as described by Mimuro *et al.* [18]. Flow cytometric analysis was then performed using a FACSCalibur flow cytometer and CellQuest software (BD Biosciences). The percentages of apoptotic cells were shown as fold increase over the respective non-infected control cells.

### Statistics

Results were expressed as mean±SEM of three independent experiments. The statistical significance of the differences was evaluated by unpaired Student's *t*-test or by analysis of variance (ANOVA) followed by Newman-Keuls' *Q*-test; significance was set at *P*<0.05. Data expressed as a percentage of control were analyzed before being normalized versus control.

## Supporting Information

Figure S1 Construction and expression into gastric epithelial cells of the GFP-CagA C-ter fusion proteins. (A) Schematic representation of the GFP-CagA C-ter fusion proteins. (B) GFP-CagA C-ter constructs expressed in AGS cells. *: shown here with the wt form; the mut one gave a virtually identical expression level and identical results (not shown). The mock vector (GFP) served as a control. Constructs in membrane (Mb) or cytosol (Cy) fractions or total cell lysate (T) were analyzed by immunoblotting (IB) using anti-GFP or anti-CagA antibodies. kDa: kilodalton. (C) AGS cells tranfected with GFP-CagA C-ter, either wt or mut, were immunoprecipitated (IP) for phosphotyrosines and blotted by anti-CagA.
(0.76 MB PDF)

Figure S2 Only transfection with GFP-CagA C-ter wt impairs VacA arrival into late endosomes of gastric epithelial cells. (A and B) VacA colocalization with the late endosomal marker LAMP1 in AGS (A) or MKN 28 (B) cells transfected with GFP or GFP-CagA C-ter, either wt or mut. After a VacA binding step of 1 h at 4°C, cells were allowed to internalize the toxin for 120 min and then were fixed. VacA (red) and LAMP1 (blue). Transfected cells (green). All the pictures shown represent single confocal sections. Scale bar: 10 µm. (C) Percentage of late endosomes (i.e., LAMP1-positive vesicles) containing VacA in either AGS or MKN 28 cells variously transfected and treated as above. Mean±SEM by extensive confocal microscopy evaluation of slides from 3 independent experiments. *: *P*<0.05 versus non-transfected cells.
(1.44 MB PDF)

Figure S3 Transfection with GFP-CagA C-ter, either wt or mut, does not alter expression of Bcl2, Bcl-xL, or Mcl1 in gastric epithelial cells. Representative blot (left) showing expression of Bcl2, Bcl-xL, and Mcl1 in AGS cells transfected with GFP or GFP-CagA C-ter, either wt or mut. Histograms (right) represent the amount of Bcl2, Bcl-xL, and Mcl1 (normalized for protein loading (α-tubulin) and shown as percentage of GFP-transfected control cells) for each transfection condition. Mean±SEM of 3 independent experiments. No statistically significant difference was found.
(0.69 MB PDF)

Figure S4 Further evidence that CagA antagonizes VacA-induced apoptosis in *H. pylori*-infected gastric epithelial cells. Apoptosis degree (shown as fold increase over the respective non-infected control cells) of AGS or MKN 28 cells infected with the wild-type CagA^+^/VacA^+^ *H. pylori* strain G27 (WT) or its isogenic mutant lacking CagA (*ΔcagA*). Mean±SEM of 3 independent experiments. *: *P*<0.05 versus control. °: *P*<0.05 versus WT.
(0.63 MB PDF)

Table S1 Primers for the construction of the fusion proteins used, and for site-directed mutagenesis of tyrosine residues of the EPIYA motifs into glycine.
(0.05 MB PDF)
