# Supplementary material for: Helicobacter pylori Counteracts the Apoptotic Action of Its VacA Toxin by Injecting the CagA Protein into Gastric Epithelial Cells
Source: PLoS Pathog. 2009 Oct 2;5(10):e1000603. doi: 10.1371/journal.ppat.1000603 (PMC2745580; doi:10.1371/journal.ppat.1000603)
Supplement: Figure S1 — Construction and expression into gastric epithelial cells of the GFP-CagA C-ter fusion proteins. (A) Schematic representation of the GFP-CagA C-ter fusion proteins. (B) GFP-CagA C-ter constructs expressed in AGS cells. *: shown here with the wt form; the mut one gave a virtually identical expression level and identical results (not shown). The mock vector (GFP) served as a control. Constructs in membrane (Mb) or cytosol (Cy) fractions or total cell lysate (T) were analyzed by immunoblotting (IB) using anti-GFP or anti-CagA antibodies. kDa: kilodalton. (C) AGS cells tranfected with GFP-CagA C-ter, either wt or mut, were immunoprecipitated (IP) for phosphotyrosines and blotted by anti-CagA. (0.76 MB PDF) [file ppat.1000603.s001.pdf]

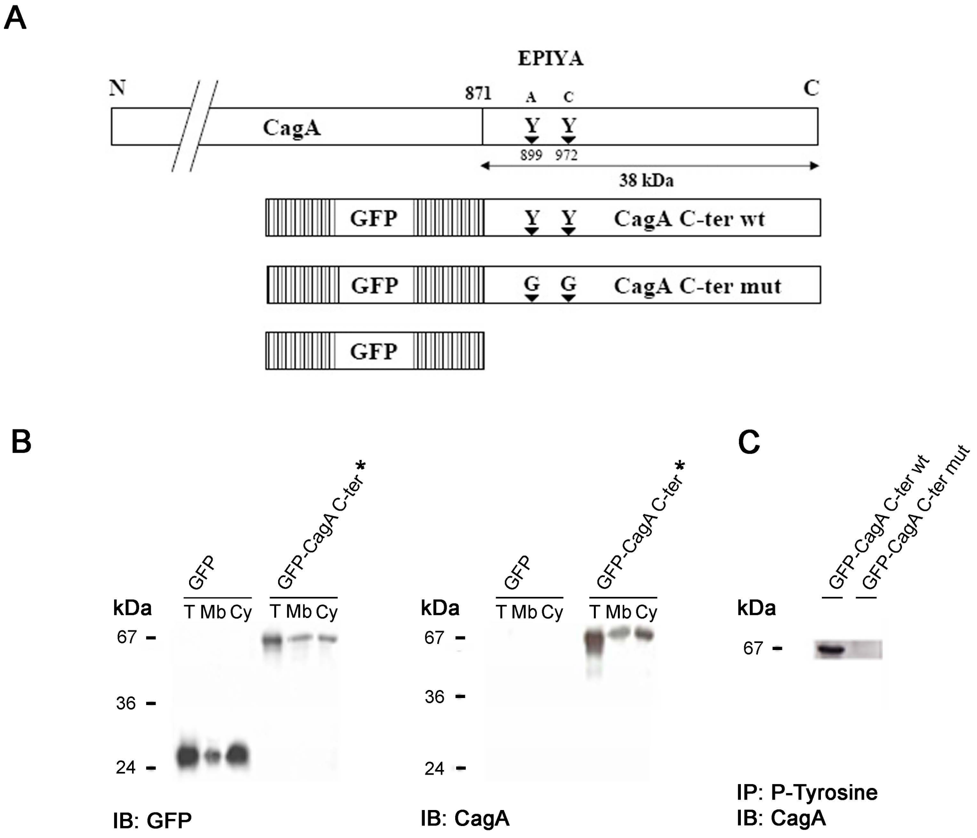

Figure S1. ***Construction and expression into gastric epithelial cells of the GFP-CagA C-ter fusion proteins.***

(**A**) Schematic representation of the GFP-CagA C-ter fusion proteins.

(**B**) GFP-CagA C-ter constructs expressed in AGS cells. *: shown here with the wt form; the mut one gave a virtually identical expression level and identical results (not shown). The mock vector (GFP) served as a control. Constructs in membrane (Mb) or cytosol (Cy) fractions or total cell lysate (T) were analyzed by immunoblotting (IB) using anti-GFP or anti-CagA antibodies. kDa: kilodalton.

(**C**) AGS cells tranfected with GFP-CagA C-ter, either wt or mut, were immunoprecipitated (IP) for phosphotyrosines and blotted by anti-CagA.
