# Supplementary material for: Helicobacter pylori Counteracts the Apoptotic Action of Its VacA Toxin by Injecting the CagA Protein into Gastric Epithelial Cells
Source: PLoS Pathog. 2009 Oct 2;5(10):e1000603. doi: 10.1371/journal.ppat.1000603 (PMC2745580; doi:10.1371/journal.ppat.1000603)
Supplement: Figure S2 — Only transfection with GFP-CagA C-ter wt impairs VacA arrival into late endosomes of gastric epithelial cells. (A and B) VacA colocalization with the late endosomal marker LAMP1 in AGS (A) or MKN 28 (B) cells transfected with GFP or GFP-CagA C-ter, either wt or mut. After a VacA binding step of 1 h at 4°C, cells were allowed to internalize the toxin for 120 min and then were fixed. VacA (red) and LAMP1 (blue). Transfected cells (green). All the pictures shown represent single confocal sections. Scale bar: 10 µm. (C) Percentage of late endosomes (i.e., LAMP1-positive vesicles) containing VacA in either AGS or MKN 28 cells variously transfected and treated as above. Mean±SEM by extensive confocal microscopy evaluation of slides from 3 independent experiments. *: P<0.05 versus non-transfected cells. (1.44 MB PDF) [file ppat.1000603.s002.pdf]

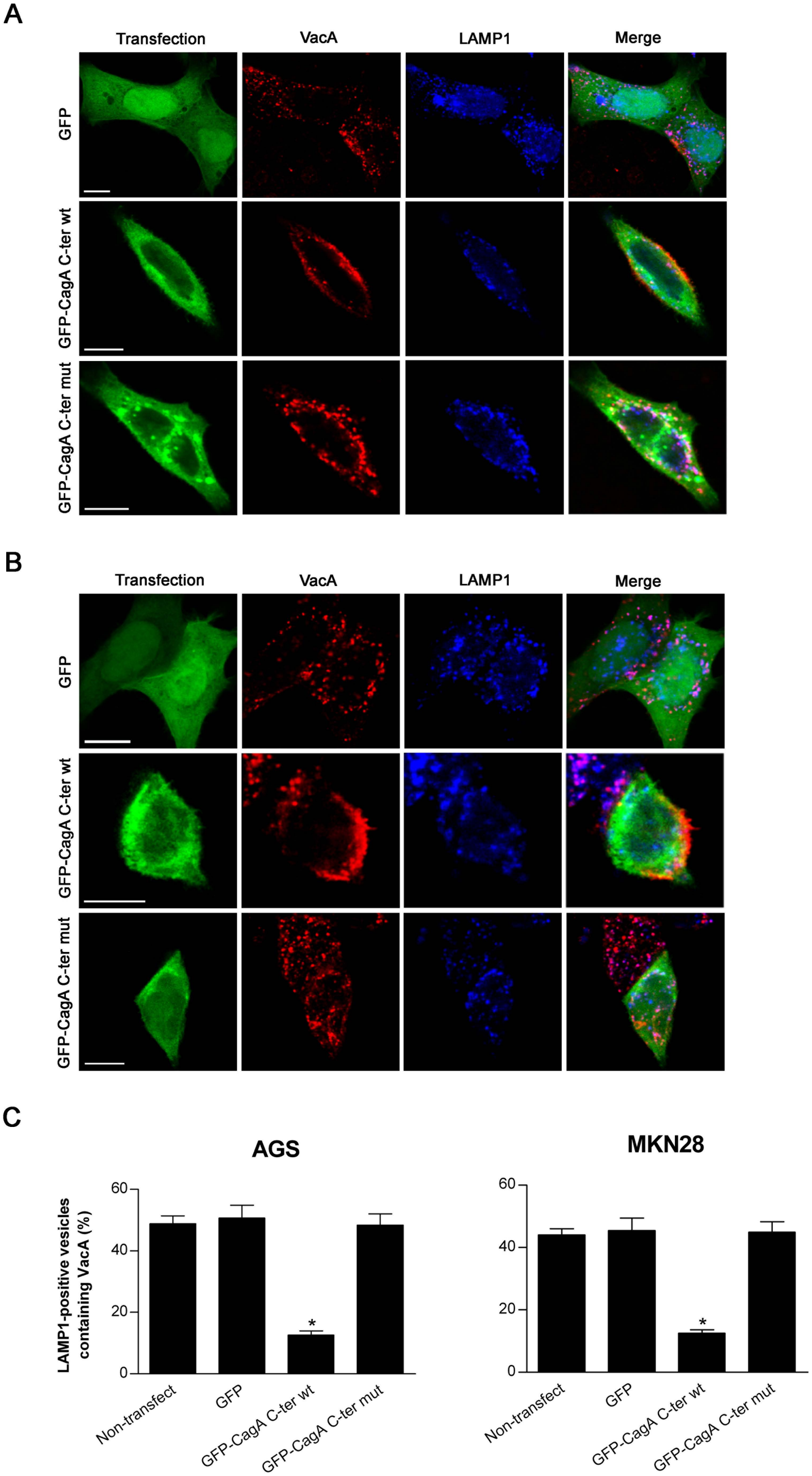

A
Transfection
VacA
LAMP1
Merge
GFP
GFP-CagA C-ter wt
GFP-CagA C-ter mut
B
Transfection
VacA
LAMP1
Merge
GFP
GFP-CagA C-ter wt
GFP-CagA C-ter mut
C
AGS
MKN28
LAMP1-positive vesicles containing VacA (%)
60
40
20
0
*
Non-transfect
GFP
GFP-CagA C-ter wt
GFP-CagA C-ter mut

Figure S2. ***Only transfection with GFP-CagA C-ter wt impairs VacA arrival into late endosomes of gastric epithelial cells.***

(**A** and **B**) VacA colocalization with the late endosomal marker LAMP1 in AGS (**A**) or MKN 28 (**B**) cells transfected with GFP or GFP-CagA C-ter, either wt or mut. After a VacA binding step of 1 h at 4°C, cells were allowed to internalize the toxin for 120 min and then were fixed. VacA (red) and LAMP1 (blue). Transfected cells (green). All the pictures shown represent single confocal sections. Scale bar: 10 μm.

(**C**) Percentage of late endosomes (i.e., LAMP1-positive vesicles) containing VacA in either AGS or MKN 28 cells variously transfected and treated as above. Mean ± SEM by extensive confocal microscopy evaluation of slides from 3 independent experiments. *: P<0.05 versus non-transfected cells.
