# Supplementary material for: Helicobacter pylori Counteracts the Apoptotic Action of Its VacA Toxin by Injecting the CagA Protein into Gastric Epithelial Cells
Source: PLoS Pathog. 2009 Oct 2;5(10):e1000603. doi: 10.1371/journal.ppat.1000603 (PMC2745580; doi:10.1371/journal.ppat.1000603)
Supplement: Figure S3 — Transfection with GFP-CagA C-ter, either wt or mut, does not alter expression of Bcl2, Bcl-xL, or Mcl1 in gastric epithelial cells. Representative blot (left) showing expression of Bcl2, Bcl-xL, and Mcl1 in AGS cells transfected with GFP or GFP-CagA C-ter, either wt or mut. Histograms (right) represent the amount of Bcl2, Bcl-xL, and Mcl1 (normalized for protein loading (α-tubulin) and shown as percentage of GFP-transfected control cells) for each transfection condition. Mean±SEM of 3 independent experiments. No statistically significant difference was found. (0.69 MB PDF) [file ppat.1000603.s003.pdf]

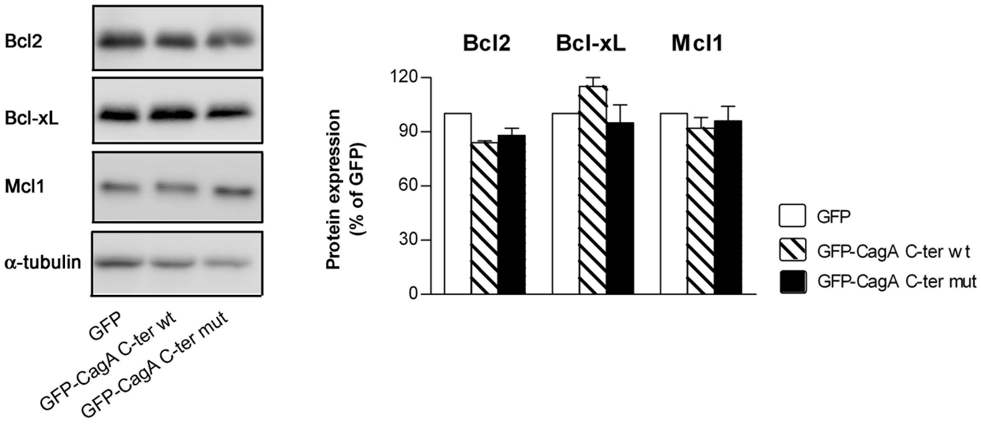

Figure S3. ***Transfection with GFP-CagA C-ter, either wt or mut, does not alter expression of Bcl2, Bcl-xL, or Mcl1 in gastric epithelial cells.***

Representative blot (left) showing expression of Bcl2, Bcl-xL, and Mcl1 in AGS cells transfected with GFP or GFP-CagA C-ter, either wt or mut. Histograms (right) represent the amount of Bcl2, Bcl-xL, and Mcl1 (normalized for protein loading (α-tubulin) and shown as percentage of GFP-transfected control cells) for each transfection condition. Mean ± SEM of 3 independent experiments. No statistically significant difference was found.
