# Supplementary material for: Helicobacter pylori Counteracts the Apoptotic Action of Its VacA Toxin by Injecting the CagA Protein into Gastric Epithelial Cells
Source: PLoS Pathog. 2009 Oct 2;5(10):e1000603. doi: 10.1371/journal.ppat.1000603 (PMC2745580; doi:10.1371/journal.ppat.1000603)
Supplement: Figure S4 — Further evidence that CagA antagonizes VacA-induced apoptosis in H. pylori-infected gastric epithelial cells. Apoptosis degree (shown as fold increase over the respective non-infected control cells) of AGS or MKN 28 cells infected with the wild-type CagA+/VacA+ H. pylori strain G27 (WT) or its isogenic mutant lacking CagA (ΔcagA). Mean±SEM of 3 independent experiments. *: P<0.05 versus control. °: P<0.05 versus WT. (0.63 MB PDF) [file ppat.1000603.s004.pdf]

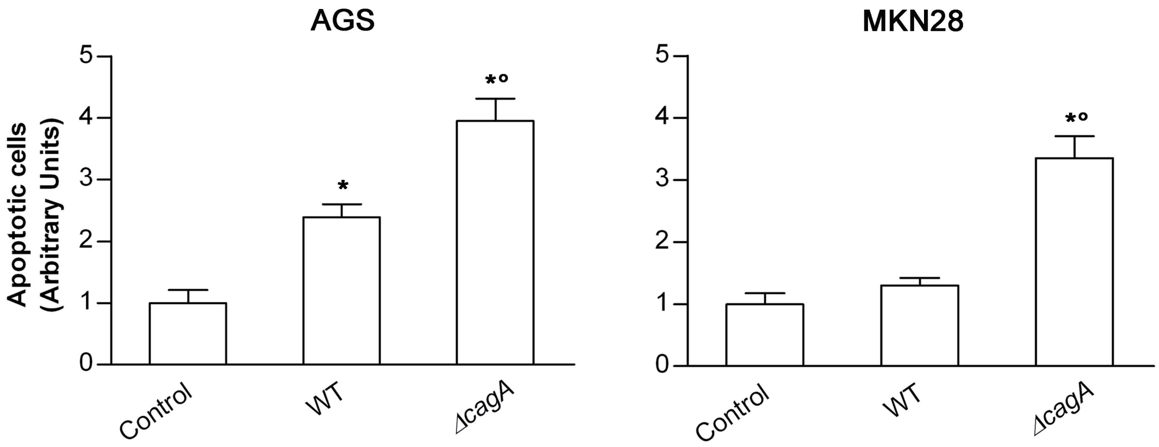

Figure S4. ***Further evidence that CagA antagonizes VacA-induced apoptosis in H. pylori-infected gastric epithelial cells.***

Apoptosis degree (shown as fold increase over the respective non-infected control cells) of AGS or MKN 28 cells infected with the wild-type CagA$^+$/VacA$^+$ *H. pylori* strain G27 (WT) or its isogenic mutant lacking CagA (Δ*cagA*). Mean ± SEM of 3 independent experiments. *: *P*<0.05 versus control. °: *P*<0.05 versus WT.
