# Supplementary material for: Helicobacter pylori Counteracts the Apoptotic Action of Its VacA Toxin by Injecting the CagA Protein into Gastric Epithelial Cells
Source: PLoS Pathog. 2009 Oct 2;5(10):e1000603. doi: 10.1371/journal.ppat.1000603 (PMC2745580; doi:10.1371/journal.ppat.1000603)
Supplement: Table S1 — Primers for the construction of the fusion proteins used, and for site-directed mutagenesis of tyrosine residues of the EPIYA motifs into glycine. (0.05 MB PDF) [file ppat.1000603.s005.pdf]

Table S1. Primers for the construction of the fusion proteins used, and for site-directed mutagenesis of tyrosine residues of the EPIYA motifs into glycine.

**CagA C-ter wt 5’**	5’ TGTACACGTCGGATATCAAGAAAGAATTG 3’
**CagA C-ter wt 3’**	5’ GCGGCCGCTTAAGATTTTTGGAAACCACCTT 3’
**Mut Y899G 5’**	5’ GCACAGAACCCATTGGTGCTAAAGTTAATAAAAAG 3’
**Mut Y899G 3’**	5’ CTTTTTATTAACTTTAGCACCAATGGGTTCTGTGC 3’
**Mut Y972G 5’**	5’ GCCCTGAACCCATTGGCGCTACGATTGATGATCTCG 3’
**Mut Y972G 3’**	5’ CGAGATCATCAATCGTAGCGCCAATGGGTTCAGGGC 3’
